# Assessing safety in the RTS,S/AS01 malaria vaccine implementation programme: an overview and comparison of methods

**DOI:** 10.1186/s12936-026-05887-z

**Published:** 2026-04-25

**Authors:** David Schellenberg, Mary Hamel, Patricia Njuguna, Eliane Furrer, Nicolas Praet, Madhava Ram Balakrishnan, Sujeet Jain, Diadié Maiga, Lode Schuerman, Cynthia G. Whitney

**Affiliations:** 1 London School of Hygiene and Tropical Medicine, London, UK; 2 Department of Immunizations, Vaccines, and Biologicals, World Health Organization, Geneva, Switzerland; 3 PATH Center for Vaccine Innovation and Access (CVIA), Nairobi, Kenya; 4 Institute of Tropical Medicine, Antwerp, Belgium; 5 Pharmacovigilance Unit, World Health Organization, Geneva, Switzerland; 6 Department of Regulation and Prequalification, World Health Organization, Geneva, Switzerland; 7 Independent Consultant, Diest, Belgium; 8 Emory University, Atlanta, USA

**Keywords:** Malaria, Vaccine, RTS,S/AS01E, Pilot, Safety assessment

## Abstract

The RTS,S/AS01 malaria vaccine implementation programme evaluated safety signals from the phase 3 trial using sentinel hospital and community-based mortality surveillance. Strengthened pharmacovigilance was used to identify rare, unanticipated signals related to vaccination. Cohort event monitoring was designed to identify rare conditions of special interest. We summarise the strengths and weaknesses of these complementary approaches to safety monitoring.

## Introduction

Following a positive scientific opinion of the RTS,S/AS01 (hereafter referred to as RTS,S) malaria vaccine by the European Medicines Agency [1] and a WHO recommendation for pilot implementation [2], the Malaria Vaccine Implementation Programme (MVIP) was designed to evaluate the feasibility, safety and impact of the RTS,S vaccine when deployed through national immunisation programmes.

Early in the MVIP, a framework for policy decision on the RTS,S malaria vaccine [3] was developed and endorsed by the Malaria Policy Advisory Group (MPAG) and Strategic Advisory Group of Experts on immunization (SAGE) to outline how the data generated by the MVIP could be used, as they became available, to inform WHO recommendations. This recognised the primary importance of establishing the safety of the vaccine and evidence of impact, and that any inadequacies in implementation could likely be addressed later.

The safety evaluation was primarily intended to investigate the signals observed in the Phase 3 trial, which involved over 15,000 children in 11 research centres across seven African countries between 2009 and 2014. In the Phase 3 trial an excess in the number of meningitis cases and the number of cerebral malaria cases was observed among RTS,S recipients when compared with children randomized to receive the comparator rabies vaccine, and a *post-hoc* analysis found an imbalance in female deaths among girls receiving the RTS,S vaccine compared with those who did not.

The pilot implementation of the malaria vaccine started in 2019 in randomly-selected areas (‘clusters’) in Ghana, Kenya and Malawi. Doses of RTS,S were given at 5, 6, 7, and 22 months of age in Malawi, and at 6, 7, 9 and 24 months of age in Ghana and Kenya. The only overlap in timing with other vaccines was at 9 months of age when children receive measles vaccine. Safety data were collected through three complementary approaches:

1. The WHO-commissioned evaluation of RTS,S introduction, referred to as the Malaria Vaccine Pilot Evaluation (MVPE). This was designed to evaluate the potential safety signals from the phase 3 trial by comparing the risk of these events in children age-eligible to receive RTS,S in implementing areas with those in non-implementing (comparison) areas [4].
2. A Phase 4 study (EPI-MAL-003) led by GSK (the vaccine manufacturer) [5], was designed to assess in implementing and comparison clusters:
  - a potential association between vaccination with RTS,S and the safety signals noted in pre-licensure study data
  - any potential association between vaccination and adverse events of special interest (Phase 4 AESI, including rare potential immune-mediated disorders [pIMDs], Table 1) and other adverse events following immunization (AEFI) leading to hospitalisation or death. These outcomes were selected as part of a general safety evaluation, and not related to specific prior safety signals.
3. Routine pharmacovigilance (PV), led by the respective Ministries of Health and Regulatory Authorities in the three pilot countries. This passive surveillance system captures and describes AEFI (including pre-specified AESI, Table 2) reported from health practitioners and the general public. Causality is assessed following the investigation of individual cases.

**Table 1:** AESI* in the GSK-sponsored phase IV study

Systemic disease and haematology	Anaphylaxis Thrombocytopenia Diabetes mellitus type I
Nerve and central nervous system	Acute disseminated encephalomyelitis Encephalitis Generalized convulsive seizure Hypotonic-hyporesponsive episode Guillain Barre Syndrome
Skin and mucous membranes, bones and joints	Henoch-Schönlein purpura Juvenile chronic arthritis Kawasaki disease Stevens-Johnson syndrome/Toxic epidermal necrolysis
Hepato-gastrointestinal and renal system	Intussusception Hepatic insufficiency Renal insufficiency

^*^Adverse events of special interest (AESI) are protocol-defined diseases corresponding to AEs that are potentially associated with RTS,S, that have historically been associated with vaccines other than RTS,S, or may hypothetically be associated with RTS,S due to the fact that this vaccine has components which are new compared to current widely used vaccines

The list of AESI was developed in collaboration with a group of paediatricians working in sub-Saharan Africa who provided advice on which additional AESI (particularly autoimmune disorders) may be of most relevance for sub-Saharan African countries. In addition, four diseases, identified as potential AE with other vaccines, were added to this list (Intussusception, Kawasaki diseases, Henoch‑Schonlein Purpura and Hypotonic Hyporesponsive Episode [HHE])

**Table 2:** AEFIs to be reported through routine PV and defined to be of special interest

Generalized	Anaphylaxis Thrombocytopenia/purpura Toxic shock syndrome
Local injection site	Cellulitis Abscess
Neurological events	Encephalo-meningeal syndrome Acute flaccid paralysis
Skin, cutaneous and joints	Stevens Johnson syndrome Toxic epidermal necrolysis Allergic hypersensitivity
Others	Major organ failure Hepatic failure Renal failure

The three approaches utilized four mechanisms to generate safety data in each of the pilot countries:

1. Sentinel hospital surveillance—included as part of the MVPE and the GSK-led Phase 4 study
2. Community-based mortality surveillance—included as part of the MVPE and the GSK-led Phase 4 study
3. Routine PV—including reporting of AEFIs and AESI following RTS,S vaccination
4. Active surveillance—included as part of the GSK-led Phase IV study, involving the enrolment of a cohort of age-eligible children and following them longitudinally for AESI at both the community level, through pre-scheduled home visits, and at primary and secondary health care facilities.

The strength of the MVIP’s safety evaluation came from the totality of the data generated through the different approaches. This paper is based on an internal document developed early in the MVIP by WHO and its partners which aimed to describe the strengths and weaknesses of the three complementary approaches to safety monitoring. We briefly review the different approaches, describe which approach(es) were used to capture which safety endpoints and how data from the different approaches informed understanding of the safety of RTS,S. Companion papers describe the strengthening of routine PV [6], the sentinel hospital and community-based mortality surveillance systems. The results of these evaluations are presented elsewhere [7, 8].

## Methods

**Fig. 1:**
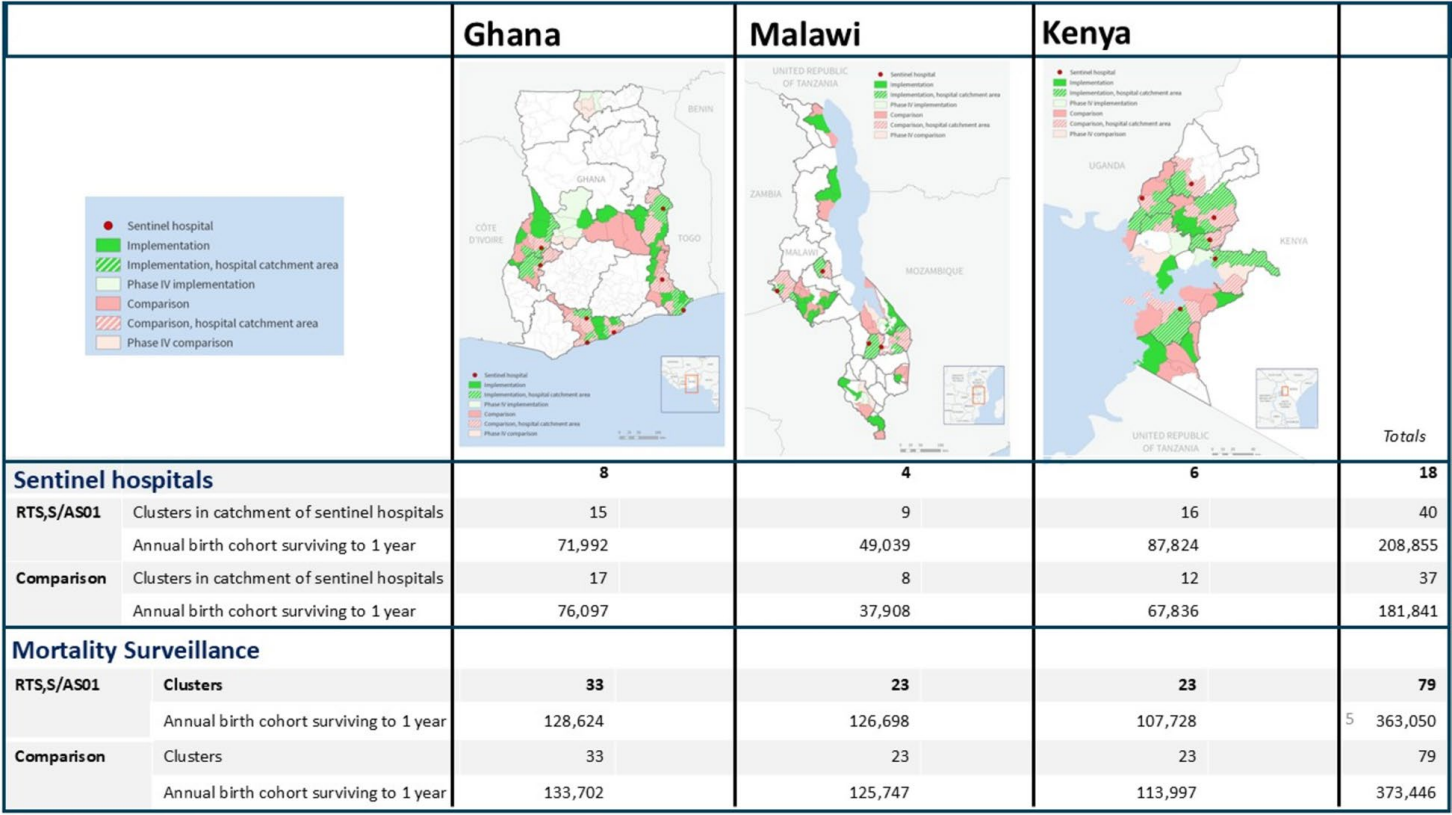
Illustration of sub-national implementation and comparison areas, covered by mortality surveillance and routine pharmacovigilance, and indicating location of sentinel hospitals and phase 4 evaluation activities

The MVIP was set up in each country to cover areas in which a total of approximately 240,0000 children were born every year (Fig. 1). Clusters were defined on the basis of administrative health service provision (in Ghana and Kenya) or immunization clinic catchment (Malawi) areas. The Ministry of Health in each country selected half of the areas, at random, to begin RTS,S implementation in 2019, and the remainder served as comparison areas, without vaccine implementation. Community-based mortality surveillance and routine pharmacovigilance covered the entire MVIP area. Sentinel hospitals covered areas with an annual birth cohort ranging from approximately 86,000 (in Malawi) to 155,000 (in Kenya), covering approximately 34–70% of the total MVPE population, split approximately equally between implementation and comparison areas. The GSK-sponsored Phase 4 study was done in geographic areas separate from the MVPE in each country.

### Sentinel hospital surveillance

The MVIP Evaluation Partners established safety surveillance systems at 18 sentinel hospitals to prospectively monitor all admissions of children potentially eligible for vaccination from implementing and comparison clusters, with an emphasis on the identification and definitive diagnosis of meningitis and cerebral malaria cases. This was the primary means to identify whether risks of meningitis or cerebral malaria were associated with the RTS,S vaccine.

The sentinel hospitals were tertiary- (or higher) level facilities where a pre-specified set of clinical data, including signs indicative of meningitis or cerebral malaria, could systematically and prospectively be documented for all admissions in the target age range.

Clinicians in the sentinel hospitals were trained to use algorithms to ensure accurate diagnosis of meningitis and cerebral malaria, involving the use of Rapid Diagnostic Tests for malaria and the collection of CSF by lumbar puncture for children with a clinical suspicion of cerebral malaria or meningitis. Investigations in local laboratories were complemented by regional WHO reference laboratories specialised in CSF examination.

Intense efforts were made to ensure the availability and use of diagnostics, and monthly review of process data helped assure complete and consistent data were available to evaluate the agreed case definitions for cerebral malaria and meningitis.

The primary safety analyses were not dependent on vaccine status. Vaccination history of admitted children, including RTS,S vaccination status, was ascertained by review of the child’s health card and, if the card was unavailable, by parental recall. Data were analysed to evaluate whether children who lived in the vaccination areas and were age-eligible to have received the RTS,S vaccine were at higher risk of meningitis or cerebral malaria compared with children who lived in the non-vaccinating, comparison areas; these data were used to determine the association of those outcomes with the RTS,S vaccine. Details of the analytical approach are presented elsewhere [9].

### Community-based mortality surveillance

The MVIP Evaluation Partners established or strengthened a network of village-based reporters to document deaths among children in the target age-group across the whole of the implementation and comparison areas (Fig. 1). In this way vaccine impact on mortality was ascertained, including any vaccine-related imbalance in mortality by sex. The vaccination history, including RTS,S vaccination status, of children who died was ascertained by review of the child’s health card, if available, or otherwise by the primary care giver’s history (potentially with supplemental information from health facility-based vaccination records). Data from the mortality surveillance were analysed to determine RTS,S vaccine impact on mortality and enabled analyses by sex to assess if there was any imbalance in sex-related mortality associated with receipt of RTS,S.

As part of both the MVPE and the Phase 4 study, investigators systematically conducted verbal autopsies on childhood deaths using the INDEPTH Standard Verbal Autopsy Questionnaire for children who died at home [10]. Because of the poor accuracy of verbal autopsy in classifying malaria or meningitis deaths [11, 12], verbal autopsy results were not used to measure the impact of the vaccine on malaria or meningitis mortality. Verbal autopsy allowed the systematic confirmation of age, vaccine status, and location, and was used to accurately identify deaths due to non-medical causes such as accidents.

### Routine PV

Pharmacovigilance (PV) refers here to the collection and analysis of AEFI spontaneously reported by health care providers and the general public. Routine PV systems have an important role in identifying signals for rare and severe adverse events, such as anaphylaxis, when their occurrence follows product administration. Such events are generally uncommon, rare or very rare and unlikely to be captured or accurately quantified during product development.

AEFI are identified through spontaneous reports generated at health facilities or in the community and submitted through the routine reporting system. The potential causal relationship between RTS,S administration and AEFI, especially the serious cases, can subsequently be investigated and classified according to standard methodology recommended by WHO [13]. PV systems may be subject to under- or over-reporting due to reporting bias. Requisites to a report being made include: (a) consideration by the patient or provider that the clinical problem may be an adverse effect of a vaccine, (b) a functioning and accessible system for reporting, and (c) willingness to report. Countries achieving the Global Vaccine Action Plan (GVAP) indicator of reporting at least 10 AEFI for 100,000 surviving infants [14] were said to have functional routine vaccine safety surveillance systems. In pilot countries this was achieved through additional technical and financial support from WHO and others, as reported elsewhere [6]. PV reports from the MVPE and Phase 4 studies comprised most of the data reviewed by the PV systems.

### Enrolment of a cohort of vaccine age-eligible children with prospective longitudinal follow-up

The GSK-led Phase IV study was set up in areas geographically distinct from the MVPE sentinel hospitals [5]. The Phase 4 study consisted of an observational cohort study including both temporal and concurrent comparisons of the occurrence of adverse events (including meningitis, AESI, deaths and other AEs leading to hospitalisation or death) and malaria (including cerebral malaria) between vaccinated and unvaccinated children living in areas with or without the RTS,S vaccine implementation. The cohort study was designed to measure all AEFI and to identify whether meningitis or cerebral malaria were causally related to RTS,S. Approximately 20,000 and 45,000 vaccine age-eligible children were enrolled at the time of initiating their routine DTP or RTS,S vaccinations, before and after RTS,S vaccine implementation, respectively. Those enrolled after the start of RTS,S implementation were split approximately equally between implementation and comparison areas.

Prospective longitudinal follow-up of enrolled children included 10 home visits during the course of approximately 4 years of follow-up and continuous monitoring of outpatient visits and hospitalisations at all primary and secondary health care facilities. Results of these evaluations have been reported elsewhere [15].

### Data monitoring

Data from all safety surveillance systems were reviewed every six months by an independent data and safety monitoring board (DSMB), convened by WHO. The DSMB comprised individuals with expertise in epidemiology, pharmacovigilance, statistics, meningitis and cerebral malaria. Representatives of the National Regulatory Authorities of the pilot countries participated in DSMB meetings, presenting the routine PV data and observing the discussion. This was intended to build understanding of the universe of safety data on RTS,S and enable informed decisions by the DSMB and NRAs.

## Discussion

The relative strengths of the safety monitoring systems to detect different safety concerns through the MVIP are illustrated in Table 3.

**Table 3:** Relative strengths of safety monitoring systems to detect different safety concerns

	Routine PV	Sentinel hospital surveillance	GSK-led Phase IV study	Community mortality surveillance
Rare, temporally related events	Yes	Yes, but underpowered	Yes	No
Cerebral malaria and meningitis signal	No	Yes	Yes	No
Mortality gender imbalance	No	No	Yes, but underpowered	Yes

Data from the routine PV systems were intended to allow the evaluation of rare, temporally related events. The system extended over the entire MVIP area, with an annual birth cohort of ~ 220,000 to ~ 260,000 per country (half of which were living in areas where the RTS,S vaccine was implemented), and was well-placed to identify rare events. Although it is recognized that AEFI surveillance is fundamental to any routine PV system, AEFI data from routine PV cannot be used to compare the safety of RTS,S between vaccinated and unvaccinated areas. This is because routine PV systems tend to capture events occurring within a few days or weeks of vaccination. RTS,S doses 1, 2 and 4 were administered at time points when no other vaccines were administered as part of the infant immunization schedule. Consequently, childhood illnesses such as meningitis, cerebral malaria, or other adverse events (AEs) that coincidentally occur between 5 and 9 months of age, or after 2 years of age, might be reported as an AEFI associated with RTS,S in areas where RTS,S is implemented. These AEs are unlikely to be reported as AEFI between 5 and 9 months of age, or after 2 years of age in the comparator areas, because no vaccines are provided at those ages. The consequence could be an impression of a causal association between the vaccine and meningitis or cerebral malaria when no such association exists, potentially resulting in withdrawal of a life-saving intervention. Hence data collected through routine PV systems are not a good basis for the comparison of the occurrence of events in vaccinated versus unvaccinated clusters. Caution is also needed when estimating the incidence of AEs as PV systems frequently under-report adverse events. The majority of AEFIs were reported from Phase 4 and sentinel hospital catchment areas, despite the much larger population under surveillance outside these areas.

Sentinel hospital surveillance provided reasonably complete capture of severe cases with detailed, prospective data collection, but was limited to the hospital catchment area, surveying admissions from an area with an annual birth cohort of ~ 390,000 across the three pilot countries. Approximately half of the children were living in areas where RTS,S was implemented, and hence the sentinel hospital surveillance allowed capture of events from areas that did not implement RTS,S. This approach therefore allows comparison of the occurrence of AEs between implementation and comparison areas. While the sentinel hospitals involved smaller areas than that covered by routine PV, they covered a substantially larger number of children than were involved in the phase 2 and 3 trials. Sentinel hospitals may therefore have been able to identify rare events. The same was true for the Phase 4 cohort event monitoring (CEM) studies which covered areas with an annual birth cohort of ~ 48,000 among the three pilot countries (half of whom were living in areas where RTS,S was implemented), but with specific efforts to detect events that are usually difficult to diagnose. Although the smallest of the MVIP-associated safety studies, the focused resources and in-depth training for identification of AESI, including pIMDs, meant that the Phase 4 study was best able to identify such rare events associated with vaccination.

The sentinel hospital surveillance approach allowed an assessment of safety outcome measures with a high level of confidence in the diagnosis and also when the outcome of interest did not necessarily occur close to the time of vaccination. This is important for the assessment of safety signals and their association with RTS,S because, in the phase 3 trial, cases of meningitis and cerebral malaria were documented throughout the 4-year period of observation. In practice, a clinician diagnosing meningitis several months after vaccination would be unlikely to attribute causality to the vaccination. However, a comparison between the risk of cases in the vaccination and non-vaccination areas can be made with statistical confidence if data are prospectively and reliably collected from both vaccination and non-vaccination areas over the same time period.

The MVIP was conducted in study areas with high mortality, covering mainly rural areas, where children who were severely ill may die before being transported to hospital. Hospital-based mortality surveillance is therefore likely to be incomplete and may be biased if, for example, care-seeking behaviour is different for boys and girls, or socioeconomic status. The Phase 4 CEM study also monitored deaths (all cause and malaria attributed deaths, and deaths attributed to an adverse event) through both sentinel hospital and active surveillance in the form of home visits, ameliorating the completeness and bias concerns. Although home visits may influence care-seeking behaviour and affect estimates of absolute rates of severe outcomes, any effects are likely to be modest, as the 10 home visits were spaced out over four years, and balanced between implementation and comparison areas. Another limitation to hospital-based mortality surveillance is the modest number of in-hospital deaths, often rendering this approach underpowered to evaluate mortality.

Community-based mortality surveillance generated data on all-cause mortality. Although verbal autopsies (VA) were used to exclude deaths due to injury from the analysis, VAs were not used for the classification of deaths due to malaria or meningitis because they generate unreliable results at the individual level [11, 12]. Hence the results of VAs were not used in the MVPE to compare cause-specific mortality endpoints between vaccinated and unvaccinated areas. Sex could reliably be assessed, resulting in confidence in the approach to evaluate a vaccine-associated imbalance in all-cause mortality by sex. The individual level follow-up of children within the GSK-led Phase 4 study provided another, more numerically limited opportunity to explore this signal.

Community mortality surveillance was not intended to detect rare fatal AEFI, unless there were clustered events temporally associated with vaccination. Historically, such events have been picked up outside of surveillance systems [16].

The MVIP sentinel hospital surveillance and community mortality surveillance required substantial, dedicated human and financial resources over multiple years. By covering large geographic areas and carefully monitoring the quality and completeness of data, robust findings were generated which informed WHO recommendations on the large-scale deployment of RTS,S/AS01.

## Conclusions

The MVIP relied on four complementary mechanisms to characterize the safety of the RTS,S vaccine in routine use. The assessment recognized the benefits of considering data from all the approaches, but also the risks should an individual system be used to characterize a particular aspect of safety when it is not designed or fit to do so. It is important that stakeholders at all levels understand the benefits and limitations of each of the approaches and the importance of utilizing each system for its intended use only. These principles, which apply equally to evaluations of other interventions, can help avoid the dissemination of misleading information. At worst, this could result in the failure to identify true causally-related safety events, or the erroneous classification of a safety signal as causally related. Either could seriously jeopardize the health of the population the interventions are intended to benefit.

## Data Availability

No datasets were generated or analysed during the current study.

## Abbreviations

AEFI: Adverse event following immunization
AESI: Adverse events of special interest
CSF: Cerebrospinal fluid
DSMB: Data and Safety Monitoring Board
EMA: European Medicines Agency
EPI: Expanded Programme on Immunization
GVAP: Global Vaccine Action Plan
GHS: Ghana Health Service
KEMRI: Kenya Medical Research Institute
MOH: Ministry of Health
MPAG: Malaria Policy Advisory Group
MVIP: Malaria Vaccine Implementation Program
MVPE: Malaria Vaccine Pilot Evaluation
NRA: National Regulatory Authority
PIMDs: Potential immune-mediated disorders
PV: Pharmacovigilance
SAGE: Strategic Advisory Group of Experts
VA: Verbal autopsies
WHO: World Health Organization
